# Two predominant MUPs, OBP3 and MUP13, are male pheromones in rats

**DOI:** 10.1186/s12983-018-0254-0

**Published:** 2018-02-23

**Authors:** Xiao Guo, Huifen Guo, Lei Zhao, Yao-Hua Zhang, Jian-Xu Zhang

**Affiliations:** 1 State Key Laboratory of Integrated Management of Pest Insects and Rodents in Agriculture, Institute of Zoology, Chinese Academy of Sciences, 1-5 Beichen West Road, Beijing, 100101 China; 2 University of Chinese Academy of Sciences, Beijing, 100049 China

**Keywords:** MUPs, Male pheromones, Female attraction, Activation of neural pathways

## Abstract

**Background:**

In rats, urine-borne male pheromones comprise organic volatile compounds and major urinary proteins (MUPs). A number of volatile pheromones have been reported, but no MUP pheromones have been identified in rat urine.

**Results:**

We used sodium dodecyl sulphate-polyacrylamide gel electrophoresis (SDS-PAGE), isoelectric focusing electrophoresis (IEF), nano-liquid chromatography-tandem mass spectrometry (nLC-MS/MS) after in gel digestion of the proteins and quantitative real-time PCR (qRT*-*PCR) and showed that the levels of two MUPs, odorant*-*binding protein 3 (OBP3) (i.e. PGCL4) and MUP13 (i.e. PGCL1), in urine and their mRNAs in liver were higher in males than in females and were suppressed by orchidectomy and restored by testosterone treatment (T treatment). We then generated recombinant MUPs (rMUPs) and found that the sexual attractiveness of urine from castrated males to females significantly increased after the addition of either recombinant OBP3 (rOBP3) or recombinant MUP13 (rMUP13). Using c-Fos immunohistochemistry, we further examined neuronal activation in the brains of female rats after they sniffed rOBP3 or rMUP13. Both rOBP3 and rMUP13 activated the accessory olfactory bulb (AOB), medial preoptic area (MPA), bed nucleus of the stria terminalis (BST), medial amygdala (MeA), posteromedial cortical amygdala (PMCo) and ventromedial nucleus of the hypothalamus (VMH), which participate in the neural circuits responsible for pheromone-induced sexual behaviours. In particular, more c-Fos-immunopositive (c-Fos-ir) cells were observed in the posterior AOB than in the anterior AOB.

**Conclusions:**

The expression of OBP3 and MUP13 was male-biased and androgen-dependent. They attracted females and activated brain areas related to sexual behaviours in female rats, suggesting that both OBP3 and MUP13 are male pheromones in rats. Particularly, an OBP excreted into urine was exemplified to be a chemical signal.

## Background

Pheromones play a crucial role in mediating socio-sexual interactions between conspecific members in rodents [1, 2]. In mice, pheromone components comprise both volatile organic compounds and non-volatile proteins, such as exocrine gland-secreted peptide 1 (ESP1) secreted by tear glands [3] and darcin (MUP20), a member of the major urinary proteins (MUPs) [4]. MUPs are low-molecular-weight (approximately 19 kDa) members of the large lipocalin family and are produced by the liver, filtered by the kidneys and secreted into the urine in some rodent species, such as mice and rats [5]. In rats, MUPs are also referred to as alpha-2u globulin (i.e. α_2*U-*_globulins) [6]. MUPs are highly polymorphic and encoded by at least 21 MUP genes on chromosome 4 in mice and approximately 9 genes and 13 pseudogenes on chromosome 5 in rats [7, 8]. MUPs are usually more prevalent in males than in females in rodents, implying a male-biased trait [9]. Rodents use voided urine to extensively mark their territories for chemosensory communication [10]. The MUPs in the deposited urine bind to and slow the release of volatile compounds, which is critical for the longevity of scent marks [11–13]. However, MUPs themselves function as chemosignals to convey socio-sexual information for conspecific receivers and thus regulate socio-sexual interactions. These two roles of MUPs are both related to urine-mediated chemical communication [1].

In rodents, pheromones acting as sexually selected traits are associated with sex, reproductive readiness and the quality of the signallers [1]. Sex pheromones are released by animals to elicit a sexual interaction with a member of the other sex of the same species and particularly mediate female mate choice for male mates in rodents [14, 15]. The identification of male pheromones has been a major focus of studies of sexual selection for many years. Currently, a few organic volatile compounds have been chemically characterized from mouse and rat urine and preputial glands and identified to be male pheromone components [12, 14, 16, 17]. Several MUPs have been characterized from male urine in mice, and some of them have been demonstrated to promote male-male territorial aggression, female attraction, conditioned place preference, or play a role in signaling individual identity [4, 18–20]. In rats, about 13 MUPs have been found in urine, including OBP3 (PGCL4), MUP13 (PGCL1), and PGCL2 [21–25]. Since the genes of the MUP family share high sequence homology, the purification of MUPs from urine and expression of a single specific protein in vitro is difficult [26], and the role in intraspecific communication of each MUP isoform in male urine, is rarely experimentally verified in rats [25, 27].

All identified mouse and rat male pheromone components, including volatile compounds and non-volatile proteins, are male-specific or more prevalent in males than in females and regulated by androgen [14, 16, 17, 28]. Male pheromone components partially or completely restore the sexual attractiveness of castrated male urine to female rodents [14, 16, 17, 28]. Male pheromones are primarily received by females via the vomeronasal organ (VNO), which then sends neuronal signals to the accessory olfactory bulb (AOB). The AOB directly targets higher centres in the brain, including the medial amygdala (MeA), bed nucleus of the stria terminalis (BST) and ventromedial nucleus of the hypothalamus (VMH), to process the sensory information [3]. The hypothalamus acts as a major source of neuroendocrine hormones that influence reproductive behaviour, and the activation of the hypothalamus by male pheromones arouses female sexual behaviour [2]. In the VNO, vomeronasal type 1 receptors (V1Rs) are specifically expressed in the apical cell layer projecting to the anterior portion of the AOB (aAOB) and are responsible for receiving signals from volatile pheromones; vomeronasal type 2 receptors (V2Rs) are mostly expressed in the basal region projecting to the posterior portion of AOB (pAOB) and receive signals from non-volatile pheromones, such as ESP1 and MUPs [29–31].

In rats, several urinary volatile compounds, such as 4-heptanone, 2-heptanone and 9-hydroxy-2-nonanone, have been definitely identified as male pheromones through a combination of chemical and behavioural studies [14, 17]. Researchers have not directly shown which single MUP isolated from rat MUPs may act as a male pheromone candidate, although a few MUPs that have been purified from urine are sexually dimorphic and positively correlated with quality, sexual attractiveness and copulatory opportunities in male rats [27, 32]. Among rat MUPs, MUP13 (also referred to as alpha-2u globulin PGCL1, UniProtKB accession number: P02761) has been isolated from rat urine and confirmed to be a kairomone, inducing fear reactions in mice [25]. OBP3 (odorant-binding protein 3, also referred to as alpha-2u globulin PGCL4, UniProtKB accession number: Q78E14), is named due to its nasal expression and odorant-binding characteristics, and has been demonstrated to be a member of the MUP family by sequence comparison [33]. Questions were raised about whether MUP13 and OBP3 function as male pheromones for conspecific communication in rats.

Volatile pheromones and MUPs are usually species-specific and widely documented in laboratory strains and wild populations of mice and rats [5, 14, 17, 34, 35]. As one of laboratory inbred rat strains, Lewis rats have clearer genetic background and lower individual variation than the outbred and wild-captured rats have, and also have higher levels of volatile pheromones than most other laboratory rat strains, providing a good model to study rat pheromones [14, 17, 35].

In the current study, we analysed MUPs in Lewis rats using sodium dodecyl sulphate-polyacrylamide gel electrophoresis (SDS-PAGE), quantitative real-time PCR (qRT*-*PCR), isoelectric focusing electrophoresis (IEF), In gel digestion and nano-liquid chromatography-tandem mass spectrometry (nLC-MS/MS), and then generated rMUPs. We further examined the activity of rMUPs by testing whether these proteins elicited behavioural and neuronal responses in female rats.

## Results

### Sexual differences in MUP levels in urine

**Fig. 1:**
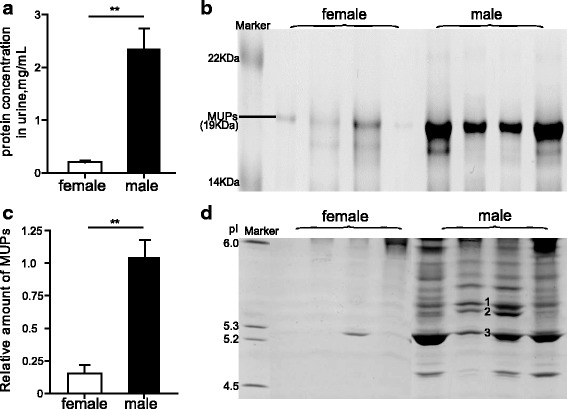
Comparison of MUP levels between males and females. **a** Concentrations of total urine proteins in males and females were assayed using the Bradford protein assay (**P < 0.01, *n* = 7, independent*-*sample *t-*test). **b** MUP levels in the urine (2 μL of diluted urine samples) from male and female Lewis rats (*n* = 7 for each sex) were detected using SDS-PAGE with a marker of molecular weight (10.5 kDa-175 kDa, CW0986S, Beijing ComWin Biotech Co., Ltd., China). One male sample was loaded on every gel as a standard to normalize the intensities of samples on different gels. The gel shown is a representative sample of four females and four males. **c** MUP abundance was quantified in SDS-PAGE gels using the ImageJ program, and the data were shown as the mean ± standard error (SE), *n* = 7 (**P < 0.01, independent-sample *t-*test). **d** Differences in MUP levels of urine (1 μL of a 1:10 dilution) between males and females (*n* = 7 for each sex) were assessed using IEF with a marker of isoelectric point (pI) in 1D gel (pI 5-6, 42,967, SERVA, Germany). The gel shown is a representative sample of four females and four males. The bands 1-3 represented the three most abundant MUPs

According to the results of the Bradford protein assay, males had approximately ten-fold higher concentrations of total urine protein than females (females, 0.20 ± 0.03 mg/mL; males, 2.30 ± 0.40 mg/mL; *P* = 0.006, *t* = 5.229, *n* = 7, independent *t-*test) (Fig. 1a). Similarly, on SDS-PAGE, MUP bands of approximately 19 kDa (between the 14 kDa and 22 kDa markers) were more abundant than the other bands in both male and female urine (Fig. 1b), and the total MUP level was significantly higher in males than in females (*P* = 0.003, *t* = 5.978, *n* = 7, independent *t-*test) (Fig. 1c). Based on the IEF results, male urine contained more MUP protein bands than female urine. In particular, bands 1-3 were the three most abundant MUPs (Fig. 1d), which respectively accounted for 10% (approximately 0.23 mg/mL), 8% (approximately 0.19 mg/mL), and 40% (approximately 0.92 mg/mL) of the total urine protein.

### Effects of castration/androgen treatment on MUP levels in urine

**Fig. 2:**
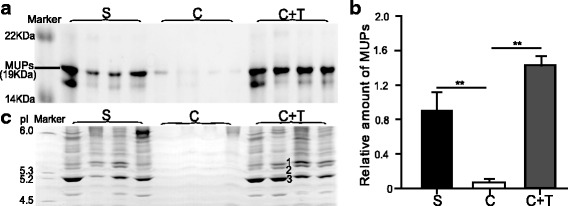
MUP levels in males were regulated by androgen. **a** SDS-PAGE showed the amount of MUPs in sham-operated, castrated and T-treated male rats (the molecular weight of marker: 10.5 kDa-175 kDa, CW0986S, Beijing ComWin Biotech Co., Ltd., China). The gel shown has 12 urine samples from three groups (2 μL of 2-fold dilution for each lane); remaining samples were on another gel which is not shown. One sham-operated male urine sample was run as a standard on every gel, and its intensity was used to normalize all values. **b** The relative abundance of MUPs on SDS-PAGE gels was analysed using the ImageJ program (mean ± SE, *n* = 7, **P < 0.01, one-way ANOVA followed by Tukey’s post hoc HSD test). **c** Each MUP in the urine samples (1 μL of a 1 in 10 dilution) from different groups was detected using IEF with a marker of pI values in 1D gel. (pI 5-6, 42,967, SERVA, Germany. S: sham-operated, C: castrated, C + T: castrated + T-treated). The bands 1-3 were the three most abundant proteins in MUPs

MUP bands were detected using SDS-PAGE (Fig. 2a). MUP levels were significantly decreased by castration but restored by T treatment (*P* < 0.001, *F* = 24.204; T-treated vs. castrated, *P* < 0.001; T-treated vs. sham-operated control, *P* = 0.065; sham-operated control vs. castrated, *P* = 0.002; *n* = 7 for each group, one-way ANOVA with Tukey’s post hoc honestly significant difference (HSD) test) (Fig. 2b). According to the IEF results, the Testosterone (T)-treated and sham-operated control groups shared a very similar IEF band pattern, and almost all single MUP bands, such as the MUP bands 1-3, were present in these two groups but were absent in the castrated group (Fig. 2c).

### Identification of MUP sequences in male urine using LC-MS/MS

**Fig. 3:**
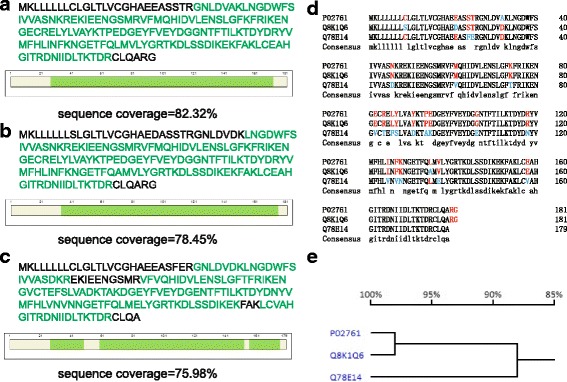
MUP sequences were identified by nLC-MS/MS in male urine; (**a**), (**b**) and (**c**) represent the sequence coverage of the three identified proteins: MUP13, Alpha-2u globulin PGCL2 and OBP3, respectively. **d** Sequence alignment of the three proteins using DANMAN software. Light blue shading shows several amino acids that differ among the three proteins. P02761, Q8K1Q6 and Q78E14 respectively represented the accession number of MUP13, Alpha-2u globulin PGCL2 and OBP3 in UniProt database. **e** Similarity analysis of the three proteins using DANMAN

The isoelectric points (pIs) of the three most intense protein bands in IEF were approximately 5.2-5.3. Mass spectrometric analysis identified the three proteins as MUP13, alpha-2u globulin PGCL2 and odorant-binding protein 3 (OBP3). The whole protein sequence was used as ExPASy entry and the sequence coverage was 82.32% (Fig. 3a), 78.45% (Fig. 3b) and 75.98% (Fig. 3c), respectively. The exclusion of the signal peptides (the first 19 amino acids) increased the sequence coverage to 92.81%, 88.95% and 86.59%, respectively. As shown in Table 1, both MUP13 and Alpha-2u globulin PGCL2 contain 181 amino acids, two more amino acids than OBP3, and the three identified proteins shared similar molecular masses (approximately 20 kDa, including the signal peptides). The pIs calculated by ExPASy-Compute pI/Mw were 5.48 for MUP13, 5.3 for Alpha-2u globulin PGCL2 and 4.7 for OBP3.

**Table 1:** List of MUPs identified in male urine using nLC-MS/MS

Accession number	Description	Score	Coverage	Unique peptides	AAs	MW (kD)	pI
P02761	Major urinary protein OS (MUP13)	926.58	82.32	3	181	20.7	5.48
Q8K1Q6	Alpha-2u globulin PGCL2	785.29	78.45	1	181	20.7	5.3
Q78E14	OBP3	90.27	75.98	9	179	20.3	4.7

The accession number was based on UniProt database. Description: the protein identified in the UniProt database. Score: ion score of the identified protein from the UniProt database. Coverage: sequence coverage calculated by dividing the number of matching amino acid residues by the total number of residues in the observed protein. Unique peptides: the number of sequences from the identified peptides that differed in at least 1 amino acid residue

*AAs* number of amino acids, *MW* molecular weight, *pI* isoelectric point

Using a sequence alignment of the three proteins by DANMAN, MUP13 and Alpha-2u globulin PGCL2 displayed high similarity (98%), with only three different amino acids, and the three proteins had a sequence similarity of approximately 88%, with 22 divergent amino acids when their signal peptides were excluded (Fig. 3d and e).

### Hepatic levels of the *Obp3* and *Mup13* mRNAs are related to sex and androgen levels

**Fig. 4:**
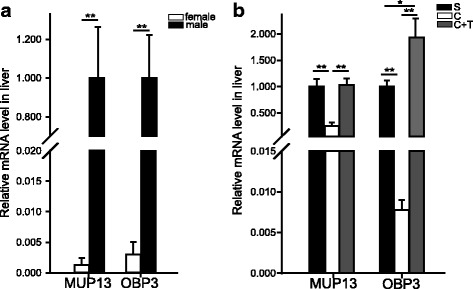
Comparison of the hepatic expression of the *Mup13* and *Obp3* mRNAs between the two sexes and among the three groups in the castration experiments. **a** Differences in *Mup13* and *Obp3* expression in female and male rats (mean ± SE, *n* = 5 for each sex, **P < 0.01, independent-sample *t-*test). **b** Expression patterns of *Mup13* and *Obp3* among groups in the castration experiment (mean ± SE, *n* = 5 for each group, *P < 0.05, ** *P* < 0.01, one-Way ANOVA followed by Tukey’s post hoc HSD test) (S: sham-operated, C: castrated, C + T: castrated + T-treated)

Female rats showed significantly lower levels of the *Obp3* (*P* = 0.009, *Z* = 2.611, *n* = 5, Mann-Whitney *U-*test) and *Mup13* (*P* = 0.009, *Z* = 2.611, *n* = 5, Mann-Whitney *U-*test) mRNAs in the liver than did their male counterparts (Fig. 4a).

Hepatic levels of both the *Obp3* and *Mup13* mRNAs were significantly downregulated in the castrated group and restored in the T-treated group (*Obp3*: *P* < 0.001, *F* = 24.092; sham-operated control vs. castrated, *P* = 0.009; T-treated vs. castrated, *P* < 0.001; T-treated vs sham-operated control, *P* = 0.013; *Mup13*: *P* = 0.001, *F* = 15.904; sham-operated control vs. castrated, *P* = 0.002; T-treated vs. castrated, *P* = 0.002; T-treated vs sham-operated control, *P* = 0.974; *n* = 5 for each group; one-way ANOVA followed by Tukey’s post hoc HSD test) (Fig. 4b).

### Behavioural tests

**Fig. 5:**
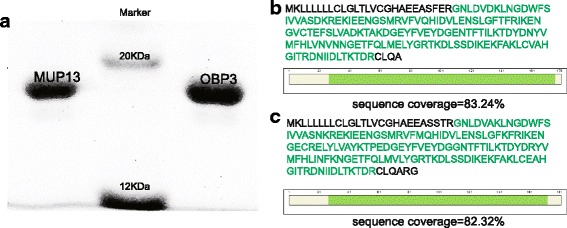
Recombinant proteins were assessed by nLC-MS/MS and SDS-PAGE. **a** Molecular weights of purified rMUP13 and rOBP3 were determined using SDS-PAGE (the molecular weight of marker: 12 kDa-120 kDa, DR201-01, Beijing TransGen Biotech Co., Ltd., China). **b** and **c** Sequence coverage of rOBP3 and rMUP13, respectively

In order to test the sexual attractiveness of OBP3 and MUP13, we produced the recombinant proteins. The rMUPs had clean bands of approximately 20 kDa in SDS-PAGE (Fig. 5a), suggesting similar molecular weight to the native MUPs, and exhibited high sequence coverage by LC-MS/MS (83.24% for rOBP3, Fig. 5b; 82.32% for rMUP13, Fig. 5c)**,** which was sufficient to confirm the expression of correct proteins.

**Fig. 6:**
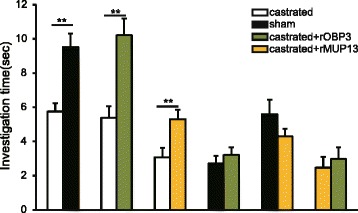
Behavioural tests were analysed by determining the time females spent investigating the samples. **a** Comparisons of the time females spent investigating samples from sham-operated and castrated males, castrated and castrated + rOBP3-treated or castrated + rMUP13-treated males, sham-operated and castrated + rOBP3-treated or castrated + rMUP13-treated males and castrated + rMUP13-treated males and castrated + rOBP3-treated males (*n* = 12 for each group, mean ± SE, **P < 0.01, *P < 0.05, Paired *t-*test or Wilcoxon signed-rank test)

Two-way choice tests revealed that females spent more time investigating the urine from males in the sham-operated control group than from males in the castrated group (*P* = 0.001, *t* = 4.852, *n* = 12, paired *t-*test). According to the IEF results, urine from intact males contained approximately 0.92 mg/mL OBP3 and 0.23 mg/mL MUP13. When rOBP3 or rMUP13 was added to urine from castrated males at the same physiological level observed in intact males, female rats showed greater attraction to treated than untreated urine from castrated males (rOBP3, *P* = 0.006, *t* = 3.434; rMUP13, *P* = 0.006, *t* = 3.366; *n* = 12, paired *t-*test). No preference was observed between urine from males in the sham-operated control group and urine from treated- castrated males (sham-operated control vs. castrated + rOBP3, *P* = 0.136, *Z* = 1.490, *n* = 12, Wilcoxon signed-rank test; sham-operated control vs. castrated + rMUP13, *P* = 0.172, *t* = 1.462, n = 12, paired *t-*test). In addition, females responded equally to the two treated urines (castrated + rOBP3 vs. castrated + rMUP13, *P* = 0.480, *Z* = 0.706, *n* = 12, Wilcoxon signed-rank test) (Fig. 6).

### Expression of c-Fos-immunoreactive (c-Fos-ir) cells in the brain of rats sniffing rMUP13 and rOBP3

**Fig. 7:**
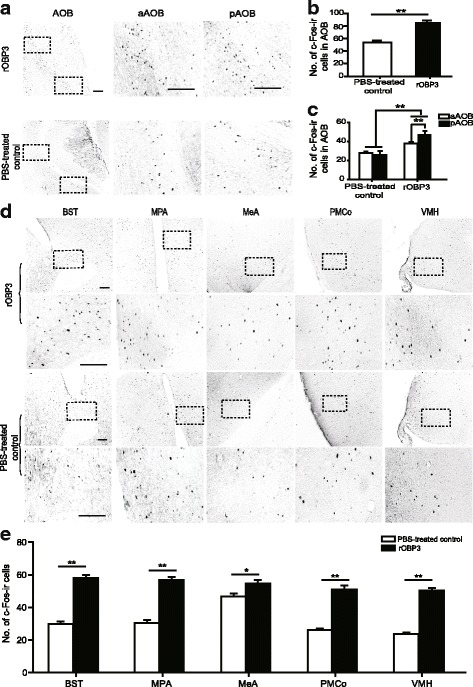
Male urine treated with rOBP3 induced c-Fos expression in females. The c-Fos-ir cells were distributed in the AOB (**a**) and higher brain areas (**d**). (**b**), (**c**) and (**e**) showed the statistical analyses of the number of c-Fos-ir cells in different brain regions (*n* = 6, mean ± SE, **P < 0.01, *P < 0.05, independent-sample *t*-test for five brain areas, AOB, aAOB and pAOB between the PBS-treated control and experimental groups; paired *t*-test or Wilcoxon signed-rank test for aAOB and pAOB in PBS-treated control or experimental rats. Scale bars = 25 μm)

**Fig. 8:**
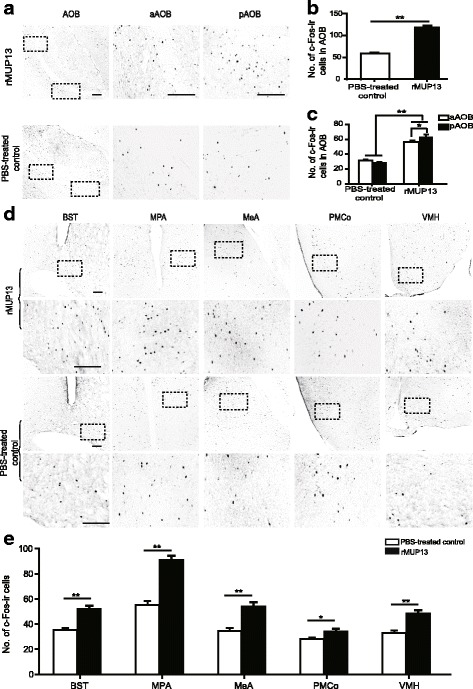
Increased c-Fos expression in females after sniffing rMUP13. Expression of c-Fos in the AOB (**a**) and five different higher brain centres (**d**); relative statistical analyses of the data presented in (**b**), (**c**) and (**e**) (*n* = 6, mean ± SE, **P < 0.01, *P < 0.05, independent-sample *t-*test for brain areas, aAOB, pAOB and AOB in PBS-treated control and treated groups, paired *t-*test or Wilcoxon signed-rank test for aAOB and pAOB from the PBS-treated control and treated groups. Scale bars = 25 μm)

The number of c-Fos-ir cells significantly increased in the AOB of females in both rOBP3- and rMUP13-exposed groups compared with those in their respective control groups (rOBP3, *P* < 0.001, *t* = 7.13; rMUP13, *P* < 0.001, *t* = 13.739; *n* = 6 for each group, independent *t-*test) (Figs. 7b and 8b). Specifically, c-Fos expression was increased in both aAOB (rOBP3, *P* < 0.001, *t* = 4.543; rMUP13, *P* < 0.001, *t* = 9.334; n = 6, independent *t-*test) and pAOB (rOBP3, *P* < 0.001, *t* = 7.621; rMUP13, *P* < 0.001, *t* = 11.532; *n* = 6, independent *t-*test) (Figs. 7c and 8c). In addition, more c-Fos-ir cells were observed in the pAOB than in aAOB in both the rOBP3- and rMUP13-exposed groups (rOBP3, *P* < 0.001, *t* = 4.039; rMUP13, *P* = 0.045, *t* = 2.084; n = 6, paired *t-*test) but not in the PBS-treated control groups (control for rOBP3, *P* = 0.505, *t* = 0.673; control for rMUP13, *P* = 0.228, *t* = 1.226; *n* = 6, paired *t-*test) (Figs. 7c and 8c).

Furthermore, females exposed to rOBP3 presented a greater number of c-Fos-ir cells in the MeA, BST, medial preoptic area (MPA), VMH and posteromedial cortical amygdala (PMCo) than the PBS-treated control females (MeA, *P* = 0.012, *t* = 2.654; BST, *P* < 0.001, *t* = 10.802; MPA, *P* < 0.001, *t* = 10.521; VMH, *P* < 0.001, *t* = 15.62; PMCo, *P* < 0.001, *t* = 9.027; *n* = 6, independent *t-*test) (Fig. 7e). Females exposed to rMUP13 also displayed more c-Fos-ir cells in these brain regions than the PBS-treated control females (MeA, *P* < 0.001, *t* = 9.877; BST, *P* < 0.001, *t* = 5.590; MPA, *P* < 0.001, *t* = 8.019; VMH, *P* < 0.001, *t* = 5.054; PMCo, *P* = 0.017, *t* = 2.534; *n* = 6, independent *t-*test) (Fig. 8e).

## Discussion

Based on our SDS-PAGE, IEF and qRT-PCR results, the total MUP concentration and the concentrations of the identified MUP13, Alpha-2u globulin PGCL2 and OBP3 proteins in voided urine exhibited a male-biased sexual dimorphism and depended on androgen in rats [5, 36–38]. Sexual dimorphism was also observed for total MUPs and darcin in mice [4, 39]. However, total MUP levels were more than ten-fold higher in males than in females in the present study, consistent with previous findings that the dichotomy is much more pronounced in rats than in mice [40, 41]. Serum testosterone exerts a stimulatory effect on MUPs, indicating that male-biased MUPs might be regulated by male hormones [36, 38]. Therefore, rat MUPs have the necessary features of chemicals used as male pheromones in rodents [15, 28].

We selected three predominant protein bands from IEF for identification by nLC-MS/MS. The three proteins were putatively identified as MUP13, Alpha-2u globulin PGCL2 and OBP3. These proteins have also been found in the voided urine of Sprague-Dawley rats, Wistar rats and wild rats [21–24, 42]. Considering the possible technical difficulties in isolating MUP members and acquiring a single specific MUP [26], we first investigated OBP3 and MUP13, for which purification has been reported [25, 33]. We generated rMUP13 and rOBP3 according to previous studies [25, 33], and studied their biological functions. The sequencing results of the clones confirmed that the genes we amplified were *mup13* and *obp3*. The identities of the two recombinant proteins were further confirmed by LC-MS-MS assay.

The combination of gel-based separation and mass measurements is often used to identify information regarding protein sequences, where proteoform-specific unique peptides analysed by high-resolution mass spectrometry are required to discriminate homologous protein superfamilies at the amino acid level [43]. IEF resolves proteins that differ in their pI values by as little as 0.01. Some studies have efficiently separated MUPs and characterized MUPs related to kinship and individual recognition in mice using IEF [10, 34]. Nineteen spots separated by two-dimensional gel electrophoresis (2-DE) were identified as Alpha-2 U*-*globulin (MUP) using matrix assisted laser desorption ionization-time of flight/time of flight (MALDI-TOF/TOF) mass spectrometry in outbred Wistar rats [21], and a unique male-specific MUP resolved by narrowed-range IEF was also characterized by MS/ MS in mouse urine [44]. Inbred Lewis rats may express fewer MUP isoforms than outbred Wistar rats, and thus, IEF is more manageable for separating MUPs from Lewis rats [45]. Although more than one MUP is contained in a single band/spot on a 2-DE gel [21], a greater number of specific unique peptides with high sequence coverage are contributed by the most abundant proteins [46] and are used for MS-based quantification to successfully identify proteins [26, 47]. In our study, the considerable sequence coverage of the matching peptides (82.32%, 78.45% and 75.98% for MUP13, Alpha-2u globulin PGCL2 and OBP3, respectively) was sufficient to demonstrate that these three bands in IEF were the predominant MUPs.

Female preferences for synthetic analogues of putative male pheromones in two- way choice tests are often utilized for the experimental verification of the sexual attractiveness and identity of male pheromones [4, 14, 17]. Volatile male mouse pheromones, such as farnesenes, hexadecanol acetate and hexadecanol, and non-volatile male mouse pheromones, including ESP1 and darcin, and volatile male rat pheromones, such as 4-heptanone, 2-heptanone and 9-hydroxy-2-nonanone, were previously identified using this methodology [3, 4, 14, 17, 28, 48]. In the current study, female rats exhibited consistent chemosensory preferences for MUP-enriched urine from males compared with that from castrated males; female rats also exhibited a preference for urine from androgen-treated castrated males compared with that from castrated males. In particular, the urine from castrated males supplemented with rMUP13 or rOBP3 showed the same attractiveness to females as urine from intact males, suggesting that both MUP13 and OBP3 in male urine aroused female attraction and were male pheromones. Besides Lewis, we have also found in Sprague-Dawley and wild-captured rats that the MUP13 and OBP3 exhibited male-biased sexual dimorphism and were associated with sexual attractiveness to female rats (unpublished data), suggesting MUP13 and OBP3 identified in Lewis male rats might be common male pheromones across strains or populations of rats.

Female sexual attraction is organized by the mutual interaction of sensory and limbic systems, particularly hypothalamic and amygdalar neurons, and produces a collection of pheromones that activate discrete inherent behaviours [1]. Male protein pheromone signals are received by the VNO and transferred to central structures that regulate the behavioural or neuroendocrine responses of female rodents [1, 3, 49, 50]. It has been found in Wistar rats that mixed MUPs induced attraction and activated neurons in the posterodorsal medial amygdala of females [27]. Here, MUP13 and OBP3 activated the characteristic neural pathways (i.e., pAOB, MeA, BST and VMH) of female rats in response to male protein pheromones, providing neural evidence for the identity of male pheromones. In particular, the activation of the hypothalamus suggested that MUP13/OBP3 stimuli may be male pheromone eliciting female sexual attraction.

MUP13 served as a male pheromone that reliably aroused female sexual preference and the defensive responses of prey [25]. Likewise, ESP1, a male pheromone in tears, not only induces sexually receptive behaviours in females but also enhances male-male aggression in mice [3, 51]. The multiple effects of pheromones on behaviour may be attributed to MeA. The MeA, receives inputs from the AOB and MOB (main olfactory bulb) and is an important centre that integrates all kinds of information and governs social behaviour. Accurate pheromone-behaviour responses, such as sex and aggression, are analysed by MeA and then controlled by the VMH [52].

The genes of MUP family are expressed in a complex sex- and tissue- specific manner [5, 37, 53, 54]. They are highly expressed in male liver and the submaxillary, lachrymal, preputial glands in both sexes and also expressed in female mammary glands [53, 54]. In rats, the mRNA of *mup13* is expressed in liver, preputial gland, and spleen, and the mRNA of obp3 was found in liver, salivary gland, submaxillary gland and nasal cavity [5]. MUPs enter urine via hepatic biosynthesis [37]. The hepatic expression of rat MUPs is under regulation of multiple hormone, and the hepatic expression is sex-dependent and under developmental control [32, 38, 55–57]. In the present study, the hepatic mRNA levels of both *mup13* and *obp3* are decreased by castration, consistent with previous findings that castration decreases the synthesis of MUPs, and the hormonal control of MUPs synthesis is associated with hepatic mRNA level [36, 38]. However, *obp3*, but not *mup13*, was significantly higher in T-treated group than in sham-operated control group, suggesting that the expressions of *obp3* was slightly different from *mup13* in response to testosterone. The mRNA of *obp3*, as well as the volatile pheromones which we reported in previous work, was overexpressed in T-treated group [14]. The total testosterone levels in serum were measured, and no significant difference was found between sham-operated control and T-treated group (1.03 ± 0.18 ng/ml vs. 0.85 ± 0.07 ng/ml, mean ± standard error, *n* = 7 for each group). It remains to be determined whether the free testosterone and other testis-related hormones are excess and related to the overexpression of *obp3* and volatiles.

Here, we first demonstrated that OBP3 and MUP13 were male pheromones in rats. They were characterized by male-biased sexual dimorphism, under androgen control and sexual attractiveness to females. Also, they could activate brain areas related to sexual behaviors in female rats. Since the members of MUP family may carry different behavioral information, it is worthwhile to investigate PGCL2 and the other MUPs in future studies.

In mice, the males of the same inbred strain share the same MUP patterns in urine; whereas the MUP patterns between strains may different, and the wild-caught mice showed wide individual variations in MUP types and amounts of MUPs [34]. Here, we found that OBP3, MUP13 and PGCL2 were consistently present in each Lewis male. Moreover, small individual variations were found within inbred Lewis males, possibly due to spontaneous mutations, stochastic and environmental events or epigenetic changes as we have evidenced for the quantitative changes of volatile pheromones among inbred C57BL/6 mice [58–60]. Previous studies on rat urinary MUPs were carried out mainly with Wistar, Sprague-Dawley and Lewis strains of rats [21–24]. However, the differences between the current work and previous work in experiment procedures and techniques make it difficult to precisely compare them. The variation in MUPs among different strains and wild populations remains to be further investigated.

## Conclusions

In our study, OBP3, MUP13 and Alpha-2u globulin PGCL2, three of the most abundant MUPs, were identified in rat urine. These three MUPs showed male-specific sexual dimorphism and androgen dependency, and may be putative pheromones. The attraction of females and the activation of neural pathways by MUP13 and OBP3 confirm that these two MUPs are both pheromones.

## Methods

### Subjects

Thirty-three male (258.3 ± 2.9 g) and 60 female (173.5 ± 2.1 g) Lewis rats at the age of 10 weeks were sexually naive and purchased from the Beijing Vital River Laboratory Animal Technology Co., Ltd., China. Males were housed individually in standard plastic rat cages (37 × 26 × 17 cm), and females were housed in groups of four animals per cage. The housing room had a reversed 14:10 h light: dark photoperiod (lights on at 19:00) and was maintained at a temperature of 23 ± 2 °C. After 2 weeks of acclimatization, the rats were used for urine collection, behavioural tests, surgical operations and immunocytochemistry. We examined the vaginal cytology to assess the oestrous stages of females, and only oestrous rats were used in all behavioral and immunohistochemical experiments.

### Surgical procedures for castration and T treatment

Twenty-one male rats were randomly selected and assigned to three groups (*n* = 7 for each group) that underwent a sham operation (sham-operated control group), bilateral orchidectomy (castrated group) and castration + T treatments, respectively [14]. For the castrated group, we anaesthetized the subjects via an intraperitoneal injection with sodium pentobarbital (40 mg/kg) and then removed the bilateral testicles following an incision of the scrotum and ligation of the blood vessels and vas deferens. For the sham-operated control group, subjects were treated with the same surgical procedures, but the bilateral gonads were not removed. For the T*-*treated group, subjects were surgically castrated and immediately received subcutaneous implantation of a silastic tubing capsule filled with crystalline testosterone (length: 20 mm, calibre: 1.57 mm, from Dow Corning Corporation, USA). The tubes were placed in the posterior scapular region of the rats, and the other two groups were implanted with empty tubes. After a 4 week postsurgical recovery period, urine was collected from all animals.

### Urine collection using metabolic cages

For urine collection, we individually caged urine donors in clean metabolic rat cages with standard rat chow and water provided and kept them continuously to collect the urine samples for 8 h daily during the dark phase of the light cycle. The urine from each metabolic cage flowed into a tube immersed in an ice box. Standard rat chow and water were freely available. Urine samples were stored at − 20 °C until use. Metabolic cages were washed thoroughly with water and sterilized between urine collections.

### Bradford protein assay

The assay reagent was prepared as follows: 200 mg of Coomassie brilliant blue (CBB) G-250 was dissolved in 100 mL of 95% ethanol and mixed with 200 mL of 85% phosphoric acid, and the solution was then diluted to 2 L with distilled water. After estimating the concentration of urine samples, each male urine sample was diluted 10-fold with 0.9% NaCl, and female urine was not diluted according to the assay’s detection limit. The urine samples and assay reagent were mixed at a 1:50 ratio, and a spectrophotometer (Beckman DU800, Beckman, USA) was used to assay the absorbance of the mixed solution at 595 nm. Bovine serum albumin (Fraction V, Genview, USA) was used to construct a standard curve. Three technical replicates were used for each urine sample and the urine protein concentrations were calculated using this standard curve [61, 62].

### SDS-page

SDS-PAGE was performed using a Mini-Protean system (Bio-Rad, USA). Each urine sample was diluted 2-fold by adding distilled water and then mixed 4:1 with 5× SDS loading-buffer (250 mM Tris-HCl (pH 6.8), 10% (*w*/*v*) SDS, 0.5% (w/v) bromophenol blue, 50% (*v*/v) glycerol, and 5% (v/v) β-mercaptoethanol). The mixed protein samples were boiled at 95 °C for 5 min, slightly spun down and then resolved in SDS-PAGE. Two microliters of the mixed samples and 4 μL of protein markers were fractionated on 15% SDS-PAGE gels at a constant voltage of 130 V. Protein gels were stained with CBB and imaged using a ChemiDoc MP system (Bio-Rad, USA). The intensity of each band was measured as the relative abundance of the protein using the ImageJ program (National Institutes of Health, USA), according to the manual (http://rsbweb.nih.gov/ij/docs/user-guide.pdf) [44, 61]. One of the male was selected as a standard and carried along on every gel to correct for differences between runs, and all values of the samples on different gels were normalized to the detected intensity of the standard sample.

### IEF

IEF was performed in a Bio-Rad model 111 Mini IEF Cell apparatus. A 5% solution of ampholytes at pH 5-6 and IEF standards with pI ranging from 3 to 10 (both from SERVA Electrophoresis GmbH, Germany) were separated on polyacrylamide gels, according to the manufacturer’s instructions. Seventy microliters of rat urine were de-salted with Zeba spin desalting columns (Pierce, USA), freeze-dried in a vacuum freeze dryer (ALPHA 1-2 LD plus, Martin Christ, Germany) and dissolved in 10 μL of deionized water. Two microliters of standards and 1 μL of a 1:10 dilution of protein samples were focused at 150 V for 15 min, 250 V for 15 min, and 450 V for 1 h, and then visualized with CBB staining [44]. The gels were imaged using a ChemiDoc MP system (Bio-Rad, USA).

### In-gel digestion

We excised each of the three predominant bands from an IEF gel of urine samples, cut the gel slice into 1 mm^3^ pieces and transferred them to 1.5-mL microcentrifuge tubes. We washed each gel piece with 200 μL of 50 mM ammonium bicarbonate (NH_4_HCO_3_) for 5 min and dehydrated the sample with 100% acetonitrile (ACN) for 15 min. We rehydrated the gel pieces by incubating them with 100 μL of 10 mM dithiothreitol at 56 °C for 30 min and then with 100 μL of 100% ACN for 3-5 min before the gel pieces were placed in 100 μL of 55 mM iodoacetamide and incubated in the dark for 20 min. After discarding the supernatant, each gel piece was washed with 100 μL of 50 mM NH_4_HCO_3_ and then with 100 μL of 100% ACN for 15 min. The gel pieces were dried in a Speed-Vac evaporator (Thermo Scientific, USA) for 20 min and digested with 13 ng/μL trypsin (sequencing grade, Promega, USA) in 50 mM NH_4_HCO_3_ overnight at 37 °C.

Supernatants were collected in fresh 1.5-mL tubes on the following day, and the remaining peptides were extracted from the gel pieces in 50 μL of a solution comprising 60% ACN, 1% trifluoroacetic acid and 39% deionized water via sonication for 10 min. The tubes were centrifuged for 30 s, and supernatants were collected. All supernatants from each band were pooled, dried in a Speed-Vac evaporator for 70 min, and mixed with 20 μL of a solution comprising 31.5% ACN, 0.1% trifluoroacetic acid (TFA) and 68.4% deionized water. The final supernatants were obtained, underwent sonication for 5 min and centrifugation, and were stored at − 20 °C until use. Unless stated otherwise, all procedures were performed at room temperature [44, 63].

### nLC-MS/MS and data analysis

The extracted peptides were analysed using an EASY-nLC 1000 system (Thermo Scientific, USA) coupled to a Q-Exactive mass spectrometer through an EASY-Spray nano-electrospray ionization source (Thermo Scientific, USA). The peptide mixtures were loaded on a trap column (Acclaim PepMap100, 75 μm × 2 cm, nano Viper C18, 3 μm, 100 Å) and separated on an analytical column (Acclaim PepMap RSLC 75 μm × 15 cm, nano Viper C18, 2 μm, 100 Å) (both Thermo Fisher Scientific, USA) using mobile phases containing solutions A (0.1% formic acid in H_2_O, chromatographic grade) and B (99.9% ACN and 0.1% formic acid). The linear gradient of buffer B increased from 0% to 30% in the first 60 min, to 95% in the next 10 min and was then sustained for 20 min at a flow rate of 300 nL/min. The eluted peptides were electrosprayed into the mass spectrometer, and raw MS data were acquired in the data-dependent acquisition mode. The Proteome Discoverer software system (version 1.4.0.288; Thermo Scientific, USA) was used to analyse the raw data and score peptides for identification. A search of the MS/MS data against the *Rattus norvegicus* database (SwissProt, 2012) was performed using the built*-*in Sequest*-*HT search engine. The mass deviation of the precursor ion, the fragment ion tolerance and the false discovery rate were set to 10 ppm, 0.05 Da and 0.01, respectively. The minimal peptide length was 6 amino acids, and two missed trypsin cleavage sites were allowed. The acrylamide modification of cysteines and methionine oxidation were set as fixed and dynamic modifications, respectively. When the search results were filtered, the matching proteins with a high peptide confidence for at least one unique peptide were considered significant [64–68]. The pIs of the identified proteins were calculated using ExPASy-Compute pI/Mw (https://web.expasy.org/compute_pi/), and DANMAN software (version 6.0, Lynnon Biosoft, USA) was used to align the protein sequences. The mass spectrometry proteomics data have been deposited to the ProteomeXchange Consortium via the PRIDE [69] partner repository with the dataset identifier PXD008341.

### qRT*-*PCR

The TRIzol method (Life Technologies, USA) was used to extract the total RNA from the rat liver, and the PrimeScript® RT reagent Kit with a gDNA Eraser (Takara Bio, Japan) was employed to reverse-transcribe cDNAs, according to the manufacturer’s instructions. Then, qRT*-*PCR was conducted with the SYBR Green SuperReal PreMix Plus Kit (Tiangen Biotech Co., Ltd., Beijing, China) and Mx3000P quantitative PCR system (Stratagene, La Jolla, CA, USA). Specific oligonucleotide primers were designed using the NCBI-Primer-BLAST online tool (https://www.ncbi.nlm.nih.gov/tools/primer-blast/index.cgi?LINK_LOC=BlastHome), and *Gapdh* was chosen as a control gene. The primers were designed as follows: *Mup13*-F: 5′- GGGAACCT CGATGTGGCTAA-3′, *Mup13*-R: 5’-GCACTCTCCATTTTCCTTAATACG-3′, *Obp3*-F: 5′-GT TGCCGACAAAACAGCAAAG-3′, *Obp3*-R: 5′-AAGGATGAAG AAATAGAT CTTGCCC-3′ and *Gapdh*-F: 5’-GACAATGAATATGGCTACA GCAAC-3′, *Gapdh*-R: 5′-TTTATTGATG GTATTCGAGAGAAGG-3′. The 20 μl reaction included 10 μL of 2× SuperReal PreMix Plus, 2 μL of cDNA template, 0.6 μL of (10 μM) of each primer, 0.2 μL of 50× ROX Reference Dye△ and 6.6 μL of RNase-free water. The amplification reaction was performed under the following conditions: denaturation for 15 min at 95 °C, followed by 40 cycles of 95 °C for 10 s, 57 °C for 20 s and 72 °C for 30 s. The temperature range for the melting curve analysis was 57 °C to 95 °C over 30 s. The data were analysed using the 2^−△△CT^ formula [70].

### Expression and purification of rOBP3 and rMUP13

The rat *Obp3* cDNA (accession number: Q78E14) was amplified by PCR from male Lewis liver using primer-F/ex/*Nde*I: 5’-GGAATTC*CATATG*GAAGAAGCTAGTTTCGA GAGAG-3′ and primer-R/ex/*Xho*I: 5’-TTCCG*CTCGAG*TCAGGCCTGGAGACAGCGATC-3′, containing *Nde*I and *Xho*I restriction enzyme sites, respectively. This amplicon was double digested with *Nde*I and *Xho*I and cloned into the pET28a expression vector (Novagen, Madison, WI, USA). The plasmid construct was transformed into *E. coli* Rosetta 2 competent cells (Novagen, USA). The positive clones were selected and sequenced using the universal primers (T7 promoter and T7 terminator primer). Analysis of the clone sequences was performed by NCBI BLAST (https://blast.ncbi.nlm.nih.gov/Blast.cgi). The rOBP3 protein was purified using HisPur Cobalt Superflow Agarose (25,229, Thermo Fisher Scientific, USA) according to the manufacturer’s instructions [33]. The rat *Mup13* cDNA (accession number: P02761) was purified using the same procedure and the following primers: F−/ex/*Nde*I:5’-GGGTTT*CATATG*CATGCAGAAGAAGCTAGTTCCACAAGAG-3′ and R−/ex/*Xho*I: 5’-CCG*CTCGAG*TCCTCGGGCCTGGAGACAG-3′. Purified rOBP3 and rMUP13 were pooled, dialyzed against 1× PBS buffer and stored at − 80 °C until use. SDS-PAGE was used to assess the purification of rOBP3 and rMUP13, and nLC-MS/MS was used to verify the identity of these two recombinant proteins.

### Two-way choice tests of female attraction to rOBP3 and rMUP13

Twenty-four female rats were used as scent recipients and randomly assigned to two groups to test the sexual attractiveness of rOBP3 and rMUP13. All behavioural tests were double blind two-way choice tests and conducted during the dark phase of the photoperiod in a separate room with a dim red light [14]. One female subject was left in the home cage while its cage mates were temporarily moved to another clean rat cage. Each subject was allowed a 15 min acclimation period prior to a trial and was used once every 4 days in a balanced order. Urine samples from 7 individuals were mixed equally for each group. The mixed samples were used to prepare scent stimuli, and rOBP3 and rMUP13 were added to urine from castrated males at the same levels observed in intact males. We painted a 2-μL scent sample on one end of the glass rod (20 cm long, 4 mm diameter) and held the other end with plastic gloves. Two glass rods painted with different scent samples were simultaneously poked through the lid of the cage in the centre of the cage and were located 1.5 cm from each other to allow the subject to freely investigate each sample. The investigation time was recorded for 3 min after the subject started to sniff or lick the rod.

### C-Fos immunohistochemistry

Each female subject was caged in a clean rat cage and housed in a separate room with an air ventilation system for 2 days of acclimation prior to use. We dissolved rMUP13 and rOBP3 in 1× PBS at concentrations equal to the levels detected in urine from intact males to prepare scent stimuli. We painted a glass slide with 20 μL of either rMUP13 or rOBP3 for the experimental group or 1× PBS for the control group (PBS-treated control group). The slide was fixed on the wire bar lid of the cage. The protein product (Fos) of the immediate early gene *c-fos* was used as a marker of neuronal activation and the number of c-Fos-immunoreactive (c-Fos-ir) cell indicated the activation levels [71]. Since the c-FOS protein synthesis follows mRNA accumulation and could be detected by immunohistochemistry at 20 to 90 min post stimulation [3, 72, 73], the subject was allowed to freely investigate the sample for 90 min.

The subject was anaesthetized with an intraperitoneal injection of sodium pentobarbital (40 mg/kg) and perfused with 100 mL of 0.9% saline through the left ventricle, followed by 100-150 mL of 4% paraformaldehyde (PFA) in 0.1 M PBS via an infusion pump (10 mL/min, Longer, UK). The brain and olfactory bulb were removed, postfixed with 4% PFA overnight, dehydrated with 30% sucrose in PBS until they fell to the bottom of the container, respectively sliced into 40 μm coronal sections and 20 μm sagittal sections using the freezing microtome (Leica, Germany), and stored in 0.1 M PBS. Brain sections including the BST, MeA, PMCo, MPA and VMH into which axons from the AOB project [3], and sections of olfactory bulb containing the AOB were selected after referencing the rat brain atlas [74]. Free-floating brain sections were selected at 80 μm intervals and serial sections of the olfactory bulb were chosen for c-Fos immunohistochemistry. Briefly, sections were blocked with 10% normal goat serum in PBS-T (2% goat serum in 0.01 M PBS with 0.5% Triton X-100) for 1 h and incubated with a primary antibody (1:2000 dilution) against c-Fos (ab190289, Abcam, UK) for two nights at 4 °C. We further incubated the sections with a biotinylated goat anti-rabbit secondary antibody (1:300) for 1 h and with Vector Elite ABC complex (both from Vector Laboratories Inc., USA) for 40 min. Sections were stained with a DAB Kit (Vector Laboratories, USA) for 2-3 min. The number of c-Fos-ir cells was counted using Image-Pro Plus 6.0 software (Media Cybernetics, Inc., USA) [75].

### Statistical analysis

We used the Kolmogorov-Smirnov test to examine the distribution of raw data and used either nonparametric tests or parametric tests in the subsequent analyses. Independent-sample *t*-tests or Mann-Whitney *U*-tests were used to determine the differences in the density of c-Fos-ir cells in each brain area between the control (PBS-treated) and MUP-stimulated groups and to test the sex-specific differences in urinary MUP levels and hepatic mRNA levels. One-way ANOVA followed by Tukey’s HSD tests were applied to examine the effect of the hormone status on MUP protein and mRNA levels. Paired *t*-tests or Wilcoxon signed-rank tests were used to determine the female preferences in the two-way choice tests and the differences in the density of c-Fos-ir cells between the aAOB and pAOB. All statistical analyses were conducted using SPSS (v18.0, SPSS Inc.). Significance was set to *P* < 0.05.

## Abbreviations

aAOB: Anterior portion of the AOB
ACN: Acetonitrile
AOB: Accessory olfactory bulb
BST: Bed nucleus of the stria terminalis
CBB: Coomassie brilliant blue
c-Fos-ir: C-Fos-immunoreactive
IEF: Isoelectric focusing electrophoresis
MeA: Medial amygdala
MPA: Medial preoptic area
MUPs: Major urinary proteins
NH_4_HCO_3_: Ammonium bicarbonate
nLC-MS/MS: Nano-liquid chromatography-tandem mass spectrometry
OBP3: Odorant*-*binding protein 3
pAOB: Posterior portion of the AOB
PMCo: Posteromedial cortical amygdala
qRT*-*PCR: Quantitative real-time PCR
rMUP13: Recombinant MUP13
RMUPs: Recombinant MUPs
rOBP3: Recombinant OBP3
SDS-PAGE: Sodium dodecyl sulphate-polyacrylamide gel electrophoresis
T treatment: Testosterone treatment
V1Rs: Vomeronasal type 1 receptors
V2Rs: Vomeronasal type 2 receptors
VMH: Ventromedial nucleus of the hypothalamus
